# Intracellular ethanol‐mediated oxidation and apoptosis in HepG2/CYP2E1 cells impaired by two active peptides from seahorse (*Hippocampus kuda* bleeler) protein hydrolysates via the Nrf2/HO‐1 and akt pathways

**DOI:** 10.1002/fsn3.2133

**Published:** 2021-01-29

**Authors:** Zhong‐Ji Qian, Mei‐Fang Chen, Jiali Chen, Yi Zhang, Chunxia Zhou, Pengzhi Hong, Ping Yang

**Affiliations:** ^1^ Shenzhen Institute of Guangdong Ocean University Shenzhen China; ^2^ Southern Marine Science and Engineering Guangdong Laboratory Zhanjiang China; ^3^ School of Chemistry and Environment College of Food Science and Technology Guangdong Ocean University Zhanjiang China; ^4^ Lengshuitan Bezirk Agricultural and Rural Bureau Yongzhou City China

**Keywords:** apoptosis, DNA damage, HepG2/CYP2E1, oxidative stress, peptide, seahorse

## Abstract

Seahorse (*Hippocampus kuda* Bleeler) are representative marine species in aquaculture, with special value of medicine and food. In this study, the protective effects of two peptides from seahorse hydrolysates (SHP‐1 and SHP‐2) against ethanol‐mediated oxidative stress in HepG2/CYP2E1 cells were investigated. Firstly, SHP‐1 and SHP‐2 presented no cytotoxicity. Compared with the ethanol‐treated groups, SHP‐1 and SHP‐2 increased cell viability in a concentration‐dependent manner. Secondly, SHP‐1 and SHP‐2 markedly reduced intracellular reactive oxygen species (ROS) generation, gamma‐glutamyltranspeptidase (GGT) activity, and tumor necrosis factor‐α (TNF‐α) levels and remarkably enhanced superoxide dismutase (SOD) and glutathione (GSH) activities. SHP‐1 and SHP‐2 also down‐regulated the expressions of GGT, bax, c‐caspase‐8/‐9/‐3, p‐Akt, p‐IκB‐α, p‐p65, p‐ERK, and p‐p38 but up‐regulated SOD, GSH, NF‐E2‐related factor 2 (Nrf2), heme oxygenase‐1 (HO‐1), and bcl‐2 levels, as revealed by Western blot analysis. Moreover, SHP‐1 and SHP‐2 increased the mitochondrial membrane potential (MMP), reduced DNA damage, and suppressed the nuclear translocation of p65. These results suggest that two peptides from seahorse hydrolysates can be considered a potential functional biomaterial and further improve the use value of seahorse in aquaculture.

## Graphical abstract

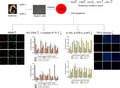

## 1 INTRODUCTION

Alcohol‐induced liver disease (ALD) is a complex process of liver damage, which results in hepatitis, vasoconstriction, hypoxia, focal necrosis, progressive fibrosis, and—finally—cirrhosis (Lands, 1995). It has been reported that ethanol‐induced oxidative stress plays a crucial role in ALD (You et al., 2017). Oxidative stress refers to an imbalance between reactive oxygen species (ROS) and the cellular antioxidant defense system (Dou et al., 2013). ROS can be generated by ethanol metabolism. ROS encompass superoxide anions, hydrogen peroxide, and hydroxyl radicals. The intracellular antioxidant defense system includes superoxide dismutase (SOD) and glutathione (GSH), which could further reduce ethanol‐induced oxidative stress (Zeng et al., 2013). Gamma‐glutamyltranspeptidase (GGT) plays a fundamental role in the maintenance of GSH homoeostasis (Kang, Qian, Ryu, Karadeniz, et al., 2012). Serum (GGT) levels are routinely examined in clinical laboratories, primarily for the diagnosis of ALD (Kang et al., 2012). Excessive intake of alcohol can cause an elevated production of ROS. Excessive ROS trigger oxidative stress, leading to mitochondrial dysfunction and DNA damage (Xing et al., 2017; Zhuang et al., 2017). ROS also play an important role in apoptosis (Su et al., 2016). Therefore, ethanol was utilized to induce oxidative injury in HepG2/CYP2E1 cells in this study.

Antioxidants are molecules that often prevent the oxidation of other molecules by being oxidized themselves (Loperena & Harrison, 2017). Antioxidants may contribute directly to decreasing oxidative cellular damage by scavenging free radicals. They may also act indirectly by inhibiting the activity or expression of free radical generating enzymes or enhancing the activity or expression of intracellular ROS‐related enzymes (Vieira et al., 2017). Recently, synthetic antioxidants (butylated hydroxytoluene [BHT], butyl hydroxy anisole [BHA], and tertiary butylhydroquinone [tBHQ]) have been widely used in food and pharmaceutical products, but these antioxidants still have some toxic and carcinogenetic effects (Finley et al., 2011; Liu et al., 2018). Thus, safer and more effective antioxidants containing natural compounds from marine biological resources are needed. Bioactive peptides are specific protein fragments that have a positive impact on a bodily function or condition and may ultimately influence human health (Zhang et al., 2019). They have a range of physiological and biochemical functions, including antimicrobial, antifatigue, antitumor, antioxidant, antihypertensive, and immunomodulatory activities (Tonolo et al., 2019). For instance, Pro‐Gly‐Trp‐Asn‐Gln‐Trp‐Phe‐Leu and Val‐Glu‐Val‐Leu‐Pro‐Pro‐Ala‐Glu‐Leu from the protein hydrolysate of the microalgae *Navicula incerta* exert antioxidant activities in HepG2/CYP2E1 cells against ethanol‐induced oxidative stress (Kang, Qian, Ryu, Karadeniz, et al., 2012). Tyr‐Gly‐Asp‐Glu‐Tyr from tilapia fish skin gelatin hydrolysates prevents HepG2 cells from experiencing alcohol‐induced damage (Chen et al., 2019).

The seahorse (*Hippocampus*), belonging to the Syngnathidae family, is a marine teleost fish widely distributed all over the world and usually found in a broad range of shallow‐water habitats, such as seagrass beds (Guo et al., 2017; Lin et al., 2008; Oh et al., 2018). Seahorse is a high‐quality material for the preparation of proteins and related products in aquaculture. *Hippocampus* is rich in proteins and essential amino acids. Previous studies have reported that a high ratio of heterocyclic or aromatic (His, Pro, Tyr, and Phe) and acidic (Glu and Asp) amino acids account for 16.14% and 20.09% of the total amino groups in *Hippocampus*, respectively (Guo et al., 2017). Moreover, recent studies have shown that amino acids, trace elements, unsaturated fatty acids, and other functional components in *Hippocampus* contribute to its hormone‐like, hematopoiesis, antiaging, antifatigue, and Ca^2+^‐blocking functions (Ryu et al., 2010a). *Hippocampus* has long been one of the essential materials in traditional Chinese medicine (Muthuramalingam et al., 2017). Because of the heavy demands for seahorses in the traditional medicine market, at least an additional 250 tons of seahorses (dry weight) are imported from Vietnam, Thailand, the Philippines, and Malaysia into China each year to produce products like seahorse wine, capsules, pills, and seahorse soup for human consumption (Lin et al., 2008). However, customers are forced to limit their utilization of seahorse products due to their high cost. It has been reported that *Hippocampus* exert a variety of biological activities, such as antioxidant, neuro‐protective, antiarthritis, and antithrombosis functions (Oh et al., 2018). For example, *Hippocampus* hydrolysates can quench free radicals and chelate metals, supporting their use as antioxidant agents (Muthuramalingam et al., 2017). Seahorse peptides exert hydroxyl free radical and DPPH scavenging activities, and have an antifatigue effect, in mice (Guo et al., 2017). The Leu‐Glu‐Asp‐Pro‐Phe‐Asp‐Lys‐Asp‐Asp‐Trp‐Asp‐Asn‐Trp‐Lys from seahorses has inhibitory effects on the collagen release in arthritis (Ryu et al., 2010b). A novel antimicrobial peptide, His‐Lys‐Pro‐Leu‐Pro, derived from *Hippocampus kuda* Bleeker, has been identified (Sun et al., 2012). However, the effects of peptides from seahorse hydrolysates (SHP) on ethanol‐induced oxidative injury in HepG2/CYP2E1 cells have not been studied.

In the present study, the protective effects of two peptides (SHP‐1, 789.88 Da, Val‐Ser‐Ile‐Ala‐Asp‐Ser‐Lys‐Ala; SHP‐2, 600.59 Da, Glu‐Asn‐Ala‐Asn‐Gly‐Pro) against ethanol‐induced oxidative stress were evaluated via their antioxidant and antiapoptosis features in HepG2/CYP2E1 cells, based on increasement of seahorse utilization and promotion of seahorse aquaculture.

## 2 MATERIALS AND METHODS

### 2.1 Materials

The two peptides from seahorse (*H. kuda* Bleeler) hydrolysates peptide (SHP) were taken from previous studies (Ryu et al., 2010a, 2010b). The HepG2/CYP2E1 cell line (HepG2 cell transfected with human CYP2E1 cDNA) was purchased from the Cell Bank of the Chinese Academy of Sciences (Shanghai, China). The Dulbecco's modified eagle's medium (DMEM) with high glucose, fetal bovine serum (FBS), 0.25% trypsin (containing EDTA), and penicillin/streptomycin were purchased from Invitrogen Corporations. The 3‐(4,5‐dimethylthiazol‐2‐yl)‐2,5‐diphenyltetrazolium bromide (MTT), dimethyl sulfoxide (DMSO), 2′,7′‐dichlorodihydrofluorescein diacetate (DCFH‐DA), and 4′,6‐diamidino‐2‐phenylindole (DAPI) were purchased from Sigma‐Aldrich. SOD and GSH detection kits were obtained from the Beyotime Institute of Biotechnology. GGT and mitochondrial membrane potential (MMP) detection kits were provided by the Nanjing Jiancheng Bioengineering Institute (Nanjing, China). The BCA protein assay kit was provided by Thermo Scientific, USA. The information on the primary and secondary antibodies used in the Western blot is shown in Table 1. The enzyme‐linked immunosorbent assay (ELISA) kit was purchased from Xinbosheng Biotechnology Co., Ltd. All other reagents were of analytical grade and were obtained from commercial sources.

**TABLE 1:** The information of the primary and secondary antibodies used in Western blot

Antibody	Source	Catalog number	Dilution	Manufacturer
GAPDH	Mouse	sc‐47724	1:500	Santa Cruz
SOD‐1	Mouse	sc‐271014	1:500	Santa Cruz
Glutathione (GSH)	Mouse	sc‐71155	1:500	Santa Cruz
GGT1	Mouse	sc‐100746	1:500	Santa Cruz
bcl‐2	Mouse	sc‐7382	1:500	Santa Cruz
bax	Mouse	sc‐20067	1:500	Santa Cruz
caspase‐8	Mouse	sc‐5263	1:500	Santa Cruz
caspase‐9	Mouse	sc‐133109	1:500	Santa Cruz
caspase‐3	Mouse	sc‐7272	1:500	Santa Cruz
c‐caspase‐3	Rabbit	9661	1:1,000	CST
Nrf2	Mouse	sc‐365949	1:500	Santa Cruz
keap1	Mouse	sc‐365626	1:500	Santa Cruz
HO‐1	Mouse	sc‐136960	1:500	Santa Cruz
Akt	Rabbit	9272	1:1,000	CST
p‐Akt	Rabbit	4060	1:1,000	CST
IκB‐α	Mouse	sc‐1643	1:500	Santa Cruz
p‐IκB‐α	Mouse	sc‐8404	1:500	Santa Cruz
p65	Mouse	sc‐8008	1:500	Santa Cruz
p‐p65	Mouse	sc‐136548	1:500	Santa Cruz
p50	Mouse	sc‐8414	1:500	Santa Cruz
p‐p50	Mouse	sc‐271908	1:500	Santa Cruz
JNK	Mouse	sc‐7345	1:500	Santa Cruz
p‐JNK	Mouse	sc‐6254	1:500	Santa Cruz
ERK 1	Rabbit	sc‐94	1:500	Santa Cruz
p‐ERK 1/2	Mouse	sc‐81492	1:500	Santa Cruz
p38	Rabbit	sc‐535	1:500	Santa Cruz
p‐p38	Mouse	sc‐166182	1:500	Santa Cruz
goat anti‐mouse IgG‐HRP	—	sc‐2005	1:5,000	Santa Cruz
goat anti‐rabbit IgG‐HRP	—	sc‐2004	1:5,000	Santa Cruz

### 2.2 Cell culture and cell viability assay

HepG2/CYP2E1 cells were cultured in DMEM with 10% FBS and 1% penicillin/streptomycin at 37°C in a humidified atmosphere containing 5% CO_2_. Cell viability was tested using an MTT assay. Cells were seeded in 96‐well plates (5 × 10^4^ cells/well) with different concentrations of SHP‐1, SHP‐2 (10, 20, 50, and 100 μM) and ethanol (0.25, 0.5, 0.75, 1, 1.25, 1.5, 1.75, and 2 M). After 24‐hr incubation, MTT (1 mg/ml, 100 μl) was added to each well and reacted in a CO_2_ incubator for 4 hr. A total of 100 μl of DMSO was added to each well, and the absorbance was detected at 570 nm. Each experiment was repeated three times.

### 2.3 Protective effects of SHP against ethanol‐induced oxidative stress

HepG2/CYP2E1 cells were grown in 96‐well plates (5 × 10^4^ cells/well), followed by incubation for 24 hr. SHP‐1 and SHP‐2 (10, 20, 50, and 100 μM) were then added to each well. After 2‐hr incubation, 0.75 M ethanol was added to each well; 24 hr later, MTT (1 mg/ml, 100 μl) was added to each well and reacted in a CO_2_ incubator for 4 hr. A total of 100 μl of DMSO was added to each well, and the absorbance was detected at 570 nm. Each experiment was repeated three times.

### 2.4 Measurement of intracellular ROS

The intracellular formation of ROS was assessed as described previously using the oxidation‐sensitive dye DCFH‐DA as the substrate. HepG2/CYP2E1 cells were grown in 24‐well plates (5 × 10^3^ cells/well). The cells’ treatment was the same as outlined in Section 2.3. Twenty‐four hours later, DCFH‐DA (10 μM, 300 μl) was added to each well and reacted for 20 min. The cells were washed with PBS three times and observed under a fluorescent inverted microscope.

### 2.5 Determination of SOD, GSH, and GGT activities

HepG2/CYP2E1 cells were seeded in 6‐well plates (5 × 10^5^ cells/ml). The cells’ treatment was the same as that outlined in Section 2.3. SOD, GSH, and GGT activities were conducted according to the manufacturer's instructions. In brief, for the SOD assay, the sample well (20 μl of sample), the blank1 well (20 μl of SOD detection buffers), and the blank2 well (40 μl of SOD detection buffers) were added to each well. Then, 160 μl of WST‐8/enzyme working fluid and 20 μl of reaction start working fluid (except for the blank2 well) were added to each well, mixed, and incubated for 30 min at 37°C. The absorbance was measured at 450 nm. SOD activity was calculated using the following equation:

$$\text{SOD}\;\left(\text{U/mg protein}\right)=\left[\left(A_{\text{blank}1}‐A_{\text{sample}}\right)/\left(A_{\text{blank}1}‐A_{\text{blank}2}\right)\right]/\left[1‐\left(A_{\text{blank}1}‐A_{\text{sample}}\right)/\left(A_{\text{blank}1}‐A_{\text{blank}2}\right)\right]\times 100\%.$$

For the GSH assay, the sample well (10 μl of sample) and the blank well (10 μl of a protein removal reagent M solution) were added to each well. Then, 150 μl of the total glutathione test solution was added. After incubation for 5 min at 25°C, NADPH (0.5 mg/ml, 50 μl) was added to each well and mixed. The absorbance was measured at 412 nm.

For the GGT assay, cell culture supernatants were collected and centrifuged. The sample well (50 μl of sample) and blank well (50 μl of ddH_2_O) were added to each well, respectively. Next, 1 ml of reagent 1 was added and reacted (37°C, 30 min). Reagent 2 was then added to each well, and reagent 1 was added to the blank well. After reacting for 5 min, the absorbance was measured at 410 nm. GGT activity was calculated using the following equation:

$$\text{GGT}\;\left(\text{U/L}\right)=\left(A_{\text{sample}}‐A_{\text{blank}}\right)\times 465.12.$$

### 2.6 Western blot analysis

HepG2/CYP2E1 cells were cultured in 6‐well plates (5 × 10^5^ cells/ml). After treatment, the cells were washed three times with PBS and lysed with RIPA containing 1% phenylmethylsulfonyl fluoride (PMSF) for 30 min on ice. Lysates were centrifuged (4°C, 6,037.2(x*g*), 20 min), and supernatant protein concentrations were determined with a BCA assay. Equal amounts (25 μg) of protein from each sample and a known molecular weight marker were loaded onto 10%, 12%, and 15% sodium dodecyl sulfate polyacrylamide gel electrophoresis (SDS‐PAGE) gels, and the proteins were separated by electrophoresis. The gels were then transferred onto nitrocellulose (NC) membranes. NC membranes were incubated with 5% skimmed milk dissolved in TBST for 4 hr. Then, the membranes were incubated with primary antibodies overnight at 4°C. After washing with TBST three times, the membranes were incubated with appropriate secondary antibodies for 2 hr and washed with TBST. The bands were visualized with an ECL system, and the band densities were optically scanned with ImageJ software.

### 2.7 Immunofluorescence assay

HepG2/CYP2E1 cells were grown on 24‐well plates (4 × 10^3^ cells/ml). After treatment, the cells were fixed with 4% paraformaldehyde (4°C, 30 min) and washed with PBS three times. The cells were then permeabilized using 0.2% Triton X‐100 for 10 min on ice and blocked in 5% BSA for 1 hr. Then, the cells were incubated with a p65 antibody dissolved in 1% BSA (1:100) over night and goat anti‐mouse IgG Dylight 488 in PBS (1:500) for 2 hr. The cells were stained with DAPI in PBS (100 ng/ml, 400 μl) for 5 min and then washed with PBS. The samples were then examined under a fluorescent inverted microscope.

### 2.8 JC‐1 staining assay

HepG2/CYP2E1 cells were seeded in 24‐well plates (4 × 10^3^ cells/ml). After treatment, the cells were washed with PBS twice, and 5,5′, 6,6‐tetrachloro‐1,1′ 3,3‐tetraethylbenzimidazolcarbocy‐anineiodide (JC‐1) was added to each well. After incubation for 20 min, the cells were washed with PBS three times and observed under a fluorescent inverted microscope.

### 2.9 Alkaline comet assay

HepG2/CYP2E1 cells were cultured in 6‐well plates. After treatment, the supernatants were removed. The cells were rinsed three times with DMEM, detached by 0.25% trypsin containing EDTA, and suspended in PBS (1 × 10^5^ cells/ml). Following the previous description (Lu et al., 2017), the first layer gel was prepared with a 0.8% normal melting point agarose gel (NMA) in PBS (0.01 M, pH 7.4). A total of 80 μl of 0.5% low melting point agarose gel (LMA) and 20 μl of the cell suspension were mixed in PBS and used as the second layer of gel. The slides were solidified (4°C, 10 min) and then immersed in a freshly prepared cold lysing solution (4°C, 2 hr). The slides were placed in an alkaline electrophoresis solution (4°C, 10 min) and then electrophoresed at room temperature (25 V, 20 min). Thereafter, the slides were washed with PBS and stained with DAPI. The comet images were taken by a fluorescent inverted microscope.

### 2.10 ELISA analysis of TNF‐α

HepG2/CYP2E1 cells were plated in 6‐well plates (5 × 10^5^ cells/ml). After treatment, cell supernatants were collected. The TNF‐α contents were determined by ELISA, which was operated according to the manufacturer's protocol.

### 2.11 Molecular docking

To further verify the interaction between SHP‐1 or SHP‐2 and SOD, bcl‐2, and bax, molecular docking was performed using C‐DOCKER in Discovery Studio 2017 R2. The structures of SHP‐1 and SHP‐2 were constructed using the Chem Office 2004 software (Cambridge Soft Co.). The protein crystal structures of SOD (ID: 1CBJ; Mi et al., 2018), bcl‐2 (ID: 1YSW; Yao et al., 2019), and bax (ID: 4ZIE; Kumar et al., 2018) were derived from the RCSB Protein Data Bank. The types of interactions between the docked proteins and the ligand were analyzed with molecular docking.

### 2.12 Statistical analysis

All experiments were performed in triplicate. Data were expressed as the mean ± *SD* (standard deviation) and were analyzed using the GraphPad Prism 5.0 software (GraphPad Prism Software Inc.). Statistical significances were determined using a one‐way ANOVA followed by Dunnett's Multiple Comparison Test. Differences were considered to be significant when *p* < .05.

## 3 RESULTS

### 3.1 Effects of SHP on HepG2/CYP2E1 cells

**FIGURE 1:**
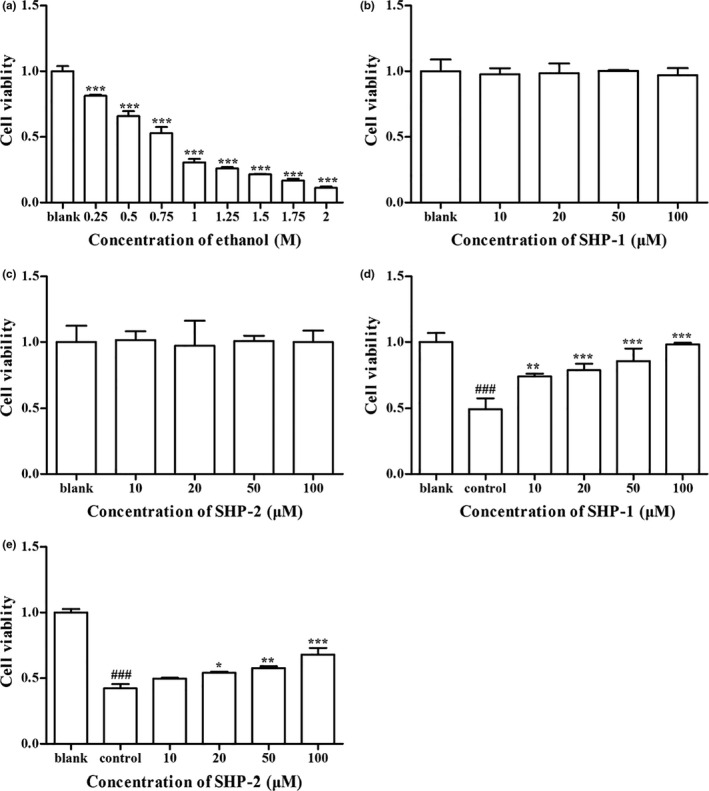
(a) Cytotoxicity of ethanol on HepG2/CYP2E1 cells. Cells were treated with ethanol (0.25, 0.5, 0.75, 1, 1.25, 1.5, 1.75, and 2 M) for 24 hr. Cell viability was measured by MTT assay. (b) Cytotoxicity of SHP‐1 on HepG2 cells. Cells were treated with SHP‐1 (10, 20, 50, and 100 μM) for 24 hr. Cell viability was detected by MTT assay. (c) Cytotoxicity of SHP‐2 on HepG2/CYP2E1 cells. Cells were treated with SHP‐2 (10, 20, 50, and 100 μM) for 24 hr. Cell viability was evaluated by MTT assay. (d) Protective effect of SHP‐1 on HepG2/CYP2E1 cells. Cells were treated with SHP‐1 for 2 hr. Then, 0.75 M ethanol was added and incubated for 24 hr. Cell viability was measured by MTT assay. (e) Protective effect of SHP‐2 on HepG2/CYP2E1 cells. Cells were treated with SHP‐2 for 2 hr. Then, 0.75 M ethanol was added and incubated for 24 hr. Cell viability was detected by MTT assay. Values are mean ± *SD* (*n* = 3). ^###^ *p* < .001 represented the control group (HepG2 cells with ethanol treatment only) vs. the blank group (HepG2 cells without treatment) and the blank group was set as 1.0. *p < .05, **p < .01, and ***p < .001 vs. the control group

The cytotoxicity of ethanol, SHP‐1, and SHP‐2 in HepG2 cells was evaluated by MTT assay. As shown in Figure 1a, the cell viability was decreased by approximately 50% in 0.75 M ethanol. Compared with the blank group, SHP‐1 and SHP‐2 (10, 20, 50, and 100 μM) showed no cytotoxicity (Figure 1b,c). The results in Figure 1d,e indicate that SHP‐1 or SHP‐2 increased cell viability in a concentration‐dependent manner in comparison with the control group.

### 3.2 Effects of SHP on intracellular ROS and SOD, GSH, and GGT

**FIGURE 2:**
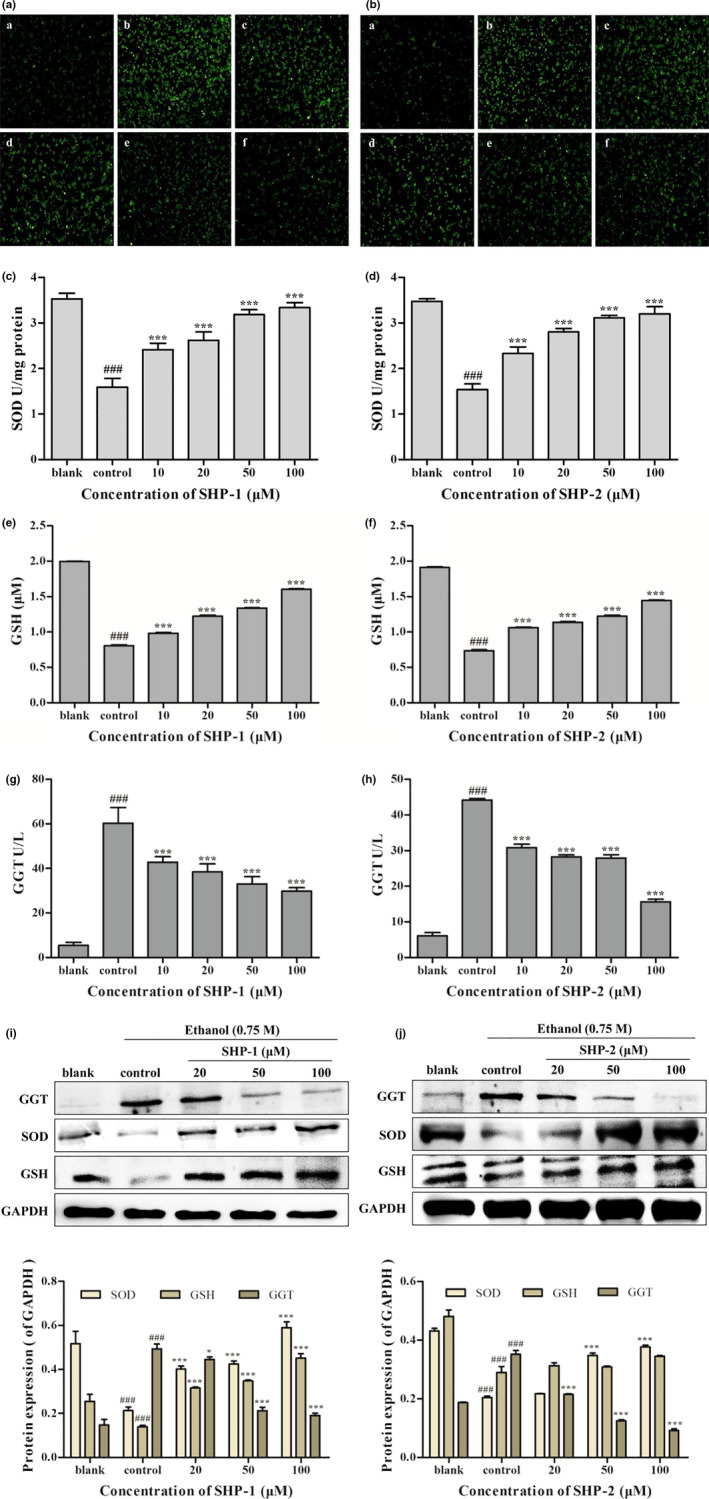
(A) Effect of SHP‐1 on intracellular ROS. Cells were treated with SHP‐1 (10, 20, 50, and 100 μM) for 2 hr. Then 0.75 M ethanol was added and incubated for 24 hr. DCFH‐DA was added and reacted for 20 min. Cells were washed with PBS three times and observed under a fluorescent inverted microscope. a, The blank group (HepG2/CYP2E1 cells without treatment); b, the control group (HepG2/CYP2E1 cells with ethanol treatment only); c–f, 10–100 μM SHP‐1. (B) Effect of SHP‐2 on intracellular ROS. a, the blank group; b, the control group; c–f, 10–100 μM SHP‐2. (C) Effect of SHP‐1 on SOD activity. Values are mean ± *SD* (*n* = 3). (D) Effect of SHP‐2 on SOD activity. Values are mean ± *SD* (*n* = 3). (E) Effect of SHP‐1 on GSH. Values are mean ± *SD* (*n* = 3). (F) Effect of SHP‐2 on GSH. Values are mean ± *SD* (*n* = 3). (G) Effect of SHP‐1 on GGT activity. Values are mean ± *SD* (*n* = 3). (H) Effect of SHP‐2 on GGT activity. Values are mean ± *SD* (*n* = 3). (I) Effect of SHP‐1 on the expressions of SOD, GSH, and GGT. Cells were treated with SHP‐1 (20, 50, and 100 μM) for 2 hr. Then, 0.75 M ethanol was added and incubated for 24 hr. SOD, GSH, and GGT levels were detected by Western blot. GAPDH was used as internal control. (J) Effect of SHP‐2 on the expressions of SOD, GSH, and GGT. ^###^ *p* < .001 represented the control group vs. the blank group. *p < .05 and ***p < .001 vs. the control group

Exposure to ethanol in HepG2/CYP2E1 cells led to ROS generation. It can be seen from Figure 2a,b that ethanol induced a significant increase in intracellular ROS generation compared with the blank group. However, SHP‐1 and SHP‐2 markedly attenuated the production of ROS (as seen by observing the fluorescence density), with respect to the control group. Moreover, as depicted in Figure 2c–h, the activities of SOD and GSH were decreased, while the activity of GGT increased after only ethanol treatment in HepG2/CYP2E1 cells. Nonetheless, the administration of SHP‐1 or SHP‐2 for 2 hr significantly enhanced SOD and GSH activities and reduced the activity of GGT. Figure 2i,j consistently showed that expressions of SOD and GSH were down‐regulated and the level of GGT was increased (but was down‐regulated in the control group). SOD and GSH levels were markedly up‐regulated, whereas the expression of GGT was significantly down‐regulated when HepG2/CYP2E1 cells were pretreated with SHP‐1 or SHP‐2.

### 3.3 Effects of SHP on the expressions of proteins in apoptosis

**FIGURE 3:**
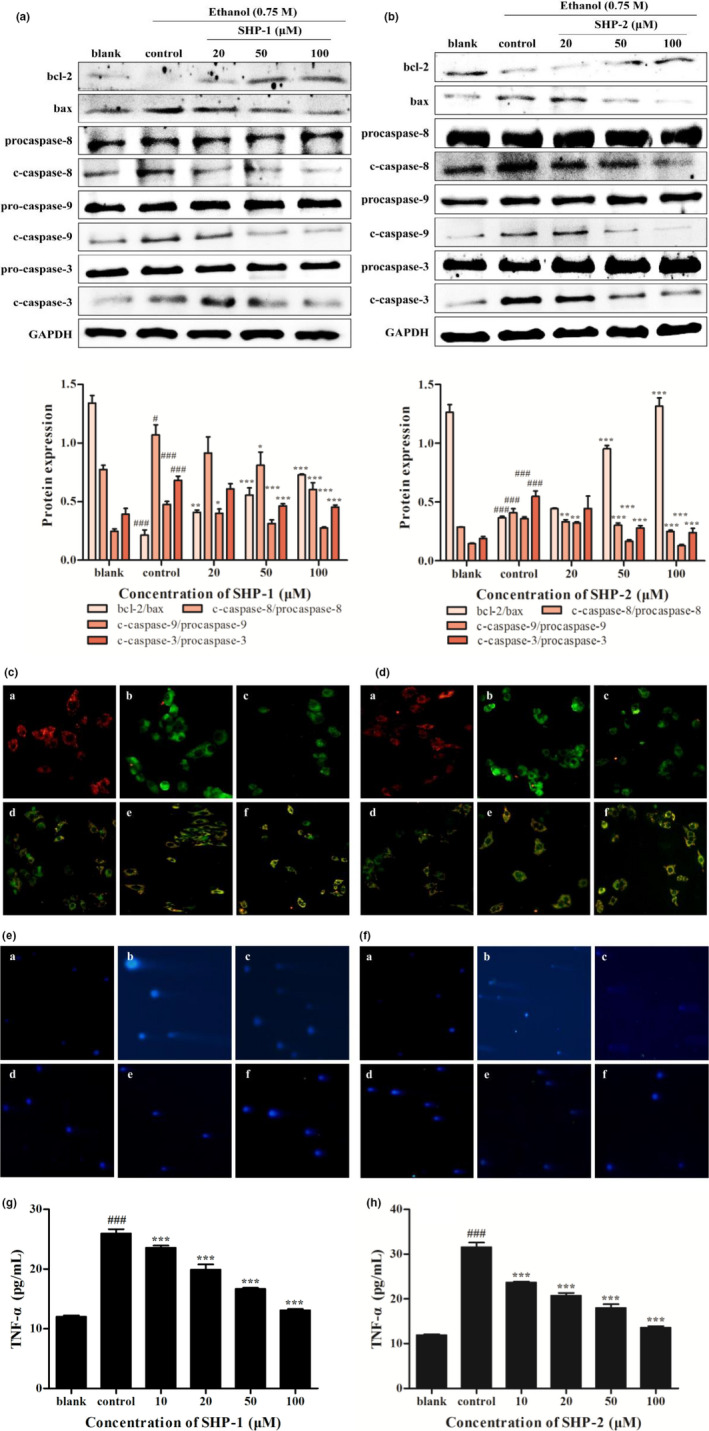
(A) Effect of SHP‐1 on the expressions of proteins in apoptosis. Cells were treated with SHP‐1 (20, 50, and 100 μM) for 2 hr. Then, 0.75 M ethanol was added and incubated for 24 hr. The expressions of proteins in apoptosis were detected by Western blot. GAPDH was used as internal control. (B) Effect of SHP‐1 on the expressions of proteins in apoptosis. (C) Effect of SHP‐1 on mitochondrial membrane potential (MMP). The treated cells were stained with JC‐1 and observed under a fluorescent inverted microscope. a, The blank group (HepG2/CYP2E1 cells without treatment); b, the control group (HepG2/CYP2E1 cells with ethanol treatment only); c–f, 10, 20, 50, and 100 μM SHP‐1. (D) Effect of SHP‐2 on MMP. a, The blank group; b, the control group; c‐f, 10, 20, 50, and 100 μM SHP‐2. (E) Effect of SHP‐1 on DNA damage. The treated cells were stained with DAPI and observed under a fluorescent inverted microscope. a, The blank group (HepG2/CYP2E1 cells without treatment); b, the control group (HepG2/CYP2E1 cells with ethanol treatment only); c–f, 10, 20, 50, and 100 μM SHP‐1. (F) Effect of SHP‐2 on DNA damage. a, The blank group; b, the control group; c‐f, 10, 20, 50, and 100 μM SHP‐2. (G) Effect of SHP‐1 on TNF‐α contents. Values are mean ± *SD* (*n* = 3). (H) Effect of SHP‐2 on TNF‐α contents. Values are mean ± S.D (*n* = 3). ^#^ *p* < .05 and ^###^ *p* < .001 represented the control group vs. the blank group. *p < .01, **p < .05, and ***p < .001 vs. the control group

The levels of apoptosis factors were evaluated using Western blot. As depicted in Figure 3b, the expressions of bax and c‐caspase‐8/‐9/‐3 were up‐regulated, while the expression of bcl‐2 was down‐regulated, after the treatment of HepG2/CYP2E1 cells with 0.75 M ethanol. Notably, the level of bcl‐2 was increased, and the expressions of bax and c‐caspase‐8/‐9/‐3 were decreased in the SHP‐1 and SHP‐2‐treatment groups. Furthermore, the expressions of procaspase‐8/‐9/‐3 had no obvious changes.

### 3.4 Effects of SHP on MMP and DNA damage

JC‐1, a fluorescence probe, has been widely used to detect MMP. JC‐1 forms aggregates with red fluorescence and high mitochondrial membrane potential. JC‐1 forms monomers with green fluorescence and low mitochondrial membrane potential. The changes from red to green fluorescence suggest that the mitochondrial membrane potential decreases. MMP collapse is a sign of ROS‐induced mitochondrial damage and a major stimulus for apoptosis (Rodenak‐Kladniew et al., 2018). Figure 3c,d indicate that the level of MMP was remarkably decreased after ethanol exposure for 24 hr. SHP‐1 or SHP‐2 administration for 2 hr markedly increased MMP compared with the control group. Furthermore, the comet assay was used to access DNA damage in HepG2/CYP2E1 cells. As displayed in Figure 3e,f, the control group had obvious comet tails compared with the blank group. However, there was a significant decrease in the comet tail lengths in the HepG2/CYP2E1 cells pretreated with SHP‐1 or SHP‐2.

### 3.5 Effects of SHP on TNF‐α contents

TNF‐α can induce apoptosis by enhancing of ROS‐mediated oxidative stress (Kang, Qian, Ryu, Karadeniz, et al., 2012; Mcclain et al., 1999). It can be seen from Figure 3g,h that ethanol treatment dramatically increased the contents of TNF‐α compared with the blank group. Meanwhile, administration of SHP‐1 or SHP‐2 from 10 to 100 μM obviously decreased the levels of TNF‐α. These findings suggest that SHP could reduce TNF‐α levels.

### 3.6 Effects of SHP on the expressions of Nrf2, Keap1, and HO‐1

**FIGURE 4:**
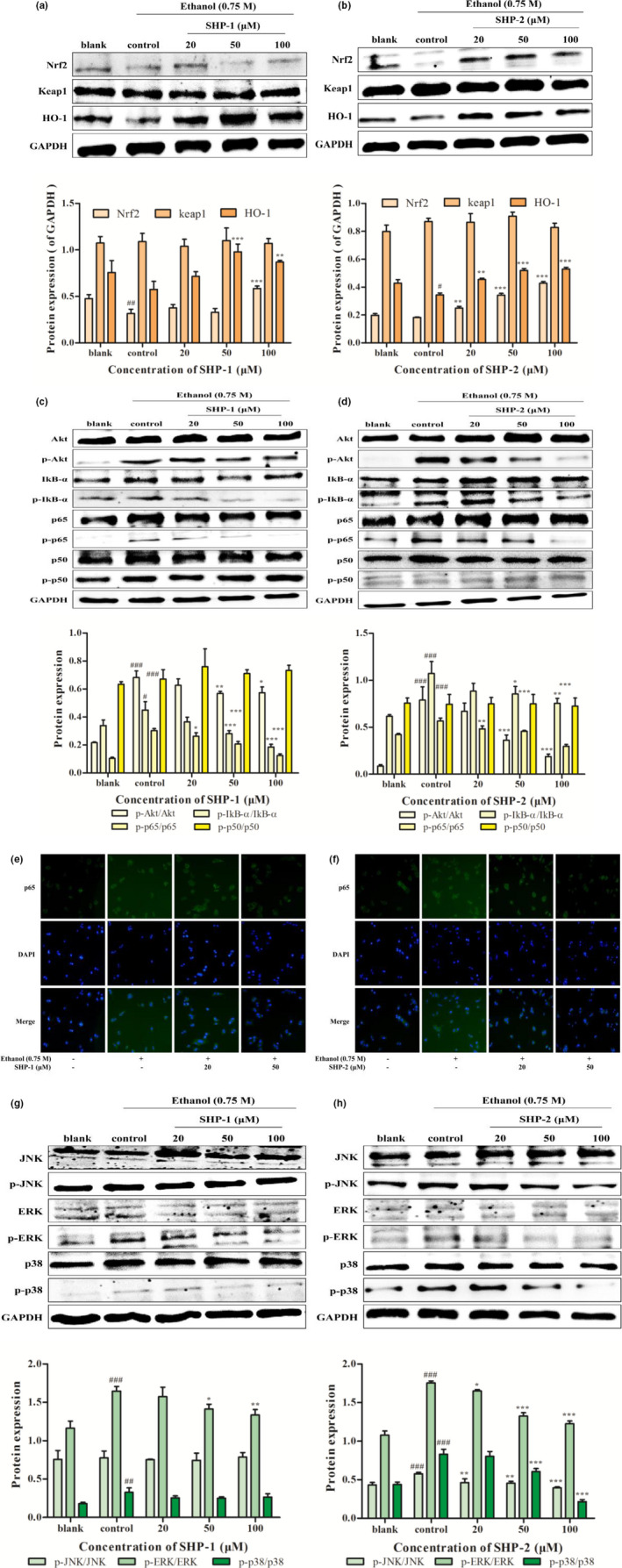
(a) Effect of SHP‐1 on the expressions of Nrf2, keap1, and HO‐1 in HepG2/CYP2E1 cells. Cells were treated with SHP‐1 (20, 50, and 100 μM) for 2 hr. Then, 0.75 M ethanol was added and incubated for 24 hr. The expressions of Nrf2, keap1, and HO‐1 were detected by Western blot. GAPDH was used as internal control. (b) Effect of SHP‐2 on the expressions of Nrf2, keap1, and HO‐1 in HepG2/CYP2E1 cells. (c) Effect of SHP‐1 on Akt and NF‐κB signaling pathways. (d) Effect of SHP‐2 on Akt and NF‐κB signaling pathways. (e) Effect of SHP‐1 on p65 nuclear translocation. Cells were treated with SHP‐1 (20 and 50 μM) for 2 hr. Then, 0.75 M ethanol was added and incubated for 24 hr. The p65 nuclear translocation was detected by immunofluorescence assay. (f) Effect of SHP‐2 on p65 nuclear translocation. (g) Effect of SHP‐1 on MAPK signaling pathway. (h) Effect of SHP‐2 on MAPK signaling pathway. ^#^ *p* < .05, ^##^ *p* < .01, and ^###^ *p* < .001 represented the control group vs. the blank group. *p < .05, **p < .01, and ***p < .001 vs. the control group

As described in Figure 4a,b, the expressions of Nrf2 and HO‐1 in HepG2 cells treated with ethanol were decreased compared with the blank group. However, after pretreatment with SHP‐1 or SHP‐2, the Nrf2 and HO‐1 levels were increased. At the same time, there were no obvious changes in the expression of keap1. These results indicate that the activation of Nrf2/HO‐1 contributed to the protective effects of SHP against ethanol‐induced oxidative stress.

### 3.7 Effects of SHP on the Akt, NF‐κB, and MAPK signaling pathways

As depicted in Figure 4c,d, the levels of p‐Akt, p‐IκB‐α, and p‐p65 were up‐regulated in the control group, whereas SHP‐1 or SHP‐2 reduced the expressions of p‐Akt, p‐IκB‐α, and p‐p65. The levels of p50 and p‐p50 showed no changes. Further, p‐ERK and p‐p38 levels were decreased in HepG2 cells in the SHP‐treatment groups (Figure 4g,h). Strikingly, SHP‐2 administration reduced the expression of p‐JNK, while the level of p‐JNK showed no obvious changes in the SHP‐1‐treatment group. These results demonstrate that SHP treatment can inhibit the Akt, NF‐κB, and MAPK signaling pathways.

### 3.8 Immunofluorescence analysis

As shown in Figure 4e,f, the fluorescence intensity of p65 increased in the cytosol of the ethanol‐treated cells compared with the blank group. Nevertheless, SHP‐1 or SHP‐2 treatment reduced the fluorescence of p65 in the cytoplasm, which further demonstrated that SHP can suppress the nuclear translocation of p65.

### 3.9 Molecular docking analysis

**FIGURE 5:**
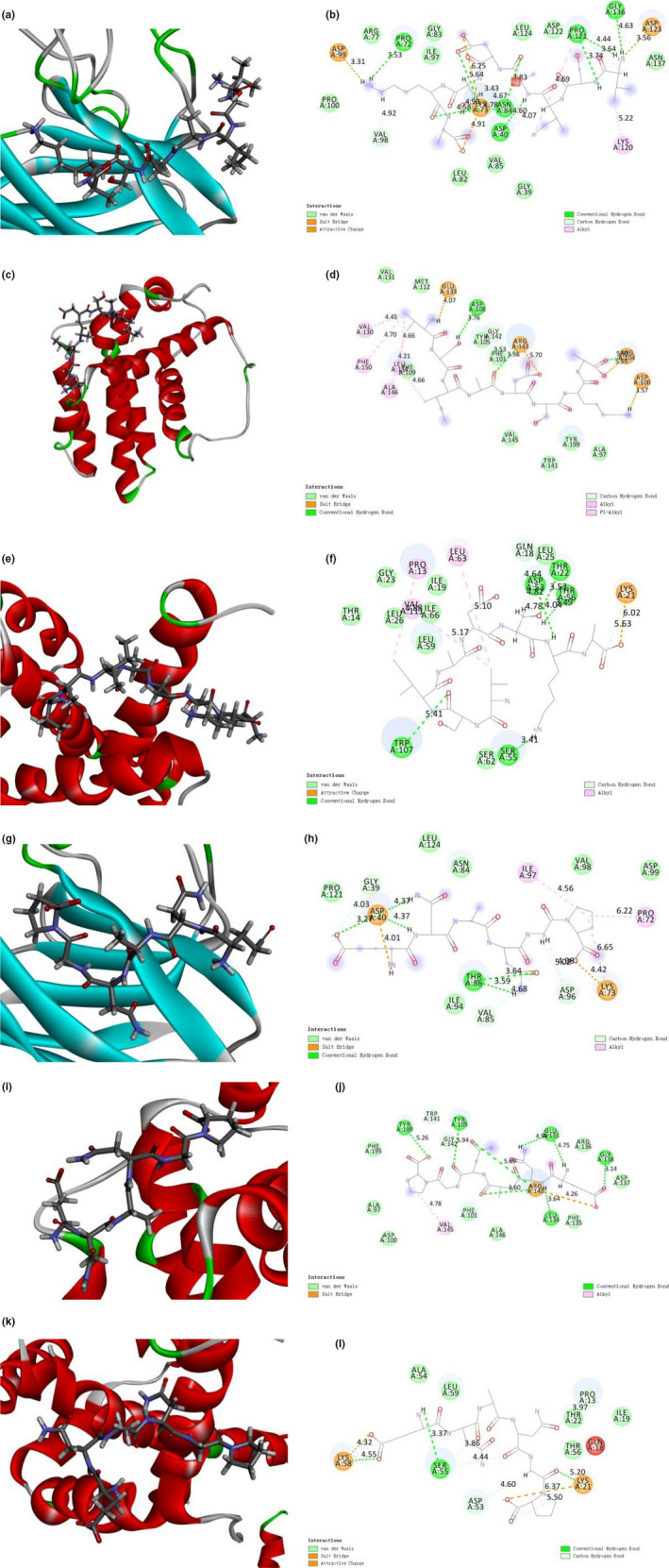
(a) 3D model and (b) 2D model of the interaction between SHP‐1 and the active site of SOD. (c) 3D model and (d) 2D model of the interaction between SHP‐1 and the active site of bcl‐2. (e) 3D model and (f) 2D model of the interaction between SHP‐1 and the active site of bax. (g) 3D model and (h) 2D model of the interaction between SHP‐2 and the active site of SOD. (i) 3D model and (j) 2D model of the interaction between SHP‐2 and the active site of bcl‐2. (k) 3D model and (l) 2D model of the interaction between SHP‐2 and the active site of bax

To further validate the interaction between SHP and SOD, bcl‐2 and bax, molecular docking was utilized. The optimum docking method is shown in Tables 2 and 3, and the optimal docking structure is illustrated in Figure 5a,c,e,g,i,k. The binding energy of SHP‐1 with SOD, bcl‐2, or bax was −7.779, −6.141, and −76.5283 kcal/moL. The binding energy between SHP‐2 and SOD, bcl‐2, and bax was −6.517, −1.637, and −62.8744 kcal/moL, respectively. Apart from these, SHP‐1 formed five hydrogen bonds with SOD (Asp40, Pro72, Asn84, Pro121, and Gly136), one hydrogen bond with bcl‐2 (Asp108), and five hydrogen bonds with bax (Thr22, Asp53, Ser55, Thr56, Trp107) in Figure 5b,d,f. One hydrogen bond (Thr86), five hydrogen bonds (Tyr105, Glu133, Leu134, Gly138, Tyr199), and one hydrogen bond (Ser55) were found between SHP‐2 and SOD, bcl‐2, and bax via hydrophobic interactions (Figure 5h,j,l), respectively. In molecular docking, SHP can tightly combine with hydrogen bond (each SHP‐1, eleven hydrogen bond; SHP‐2, seven hydrogen bond) leading to the active suppression of SOD, bcl‐2, and bax. These results are consistent with the results of Western blotting, indicating that the structure of SHP may be suitable for further exploitation leading to a compound with antioxidant activity and antiapoptosis activity potential.

**TABLE 2:** SHP‐1 docking with and SOD, bcl‐2, and bax

Docking method	Ligand	Receptor	‐CDOCKER_INTERACTION_ENERGY (kcal/moL)
1	SHP‐1	SOD	7.779
2	SHP‐1	SOD	6.449
3	SHP‐1	bcl‐2	6.141
4	SHP‐1	bcl‐2	2.533
5	SHP‐1	bax	76.5283
6	SHP‐1	bax	76.2325
7	SHP‐1	bax	74.4497

**TABLE 3:** SHP‐2 docking with and SOD, bcl‐2, and bax

Docking method	Ligand	Receptor	‐CDOCKER_INTERACTION_ENERGY (kcal/moL)
1	SHP‐2	SOD	6.517
2	SHP‐2	SOD	5.966
3	SHP‐2	bcl‐2	1.637
4	SHP‐2	bcl‐2	0.972
5	SHP‐2	bax	62.8744
6	SHP‐2	bax	61.4875
7	SHP‐2	bax	60.3284

## 4 DISCUSSION

The toxic effect of ethanol in the liver has been widely studied. Human hepatocellular carcinoma (HepG2) cells are known to metabolize ethanol nonoxidatively to fatty acid ethyl esters (FAEEs). Also, due to their many genotypic and phenotypic similarities to human hepatocytes, HepG2 cells, such as hepatic alcohol dehydrogenase (ADH) and CYP2E1, are often used for a variety of drug metabolism and toxicity studies (Kang, Qian, Ryu, Karadeniz, et al., 2012). In the present study, HepG2/CYP2E1 cells were treated with ethanol to cause oxidative stress‐induced injury and to evaluate the protective effects of SHP‐1 and SHP‐2. In general, cell viability represents the toxicity degree of toxicants, which is the most important index in a cytotoxicity test (Li et al., 2015). The MTT assay showed that ethanol treatment decreased cell viability (Figure 1a), which agrees with the previous study (Neuman et al., 1993). SHP‐1 or SHP‐2 (10–100 μM) presented no toxicity in HepG2/CYP2E1 cells and dose‐dependently enhanced cell viability compared with the group treated only with ethanol (Figure 1b–e). These results suggest that SHP‐1 and SHP‐2 can protect HepG2/CYP2E1 cells from oxidative damage. However, the mechanism remains unclear.

Alcohol has been shown to promote liver injury through an increased formation of ROS (Chandrasekaran et al., 2012). ROS play a complex role in ALD. Low levels of ROS normally stimulate cell proliferation, while excessive ROS promote irreparable cell damage leading to cell death (Rodenak‐Kladniew et al., 2018). In this study, pretreatment with SHP‐1 or SHP‐2 for 2 hr reduced the production of ROS in HepG2/CYP2E1 cells (Figure 2a,b). The hepatocellular antioxidant defense system mainly includes SOD and GSH, which play a critical role in regulating cellular ROS to maintain a dynamic balance of ROS production and elimination (Li et al., 2015). As mentioned previously, SOD converts superoxide radicals to hydrogen peroxide and oxygen (Zhuang et al., 2017). GSH, a polypeptide antioxidant, is mainly created and metabolized in the liver and plays an important role in protecting against injuries by scavenging ROS in several tissues and cell lines (Yarahmadi et al., 2017). The decrease in GSH levels has been suggested as one of the primary mechanisms of t‐BHP‐induced toxicity in liver cells (Kang, Qian, Ryu, Karadeniz, et al., 2012). GGT levels increase in response to an exposure to a variety of drugs and alcohol, contributing to the diagnosis of ALD (Kang, Qian, Ryu, Kim, et al., 2012). This may be mediated via oxidative stress, with resultant reductions in GSH levels. The previous report showed that the treatment of seahorse *H. abdominalia* hydrolysates by alcalase (SHAH) protected HUVECs against oxidative stress‐mediated injury (Oh et al., 2018). It was observed that SHP‐1 and SHP‐2 increased the activities of SOD and GSH and decreased the activity of GGT in Figure 2c–h. Meanwhile, the expressions of SOD and GSH were up‐regulated, and the level of GGT was down‐regulated (Figure 2i,j). These results reveal that oxidative stress may be the primary mechanism of cell death in HepG2/CYP2E1 cells.

High levels of ROS trigger tumor cell apoptosis (Dasgupta et al., 2019). Apoptosis is a process regulated by a series of enzymes and genes under physiological or pathological conditions (Wang et al., 2017). It is characterized by specific morphological changes, such as a condensation of chromatin, a loss of microvilli, blebbing formation, and the appearance of apoptotic bodies (Wang et al., 2013). Apoptosis may be activated by an intrinsic or extrinsic pathway. Apoptosis is regulated by various molecules, such as the bcl‐2 family and cysteinyl aspartate‐specific proteinase (caspase) family proteins (Li et al., 2015; Ye et al., 2016). The Bcl‐2 family includes the antiapoptosis protein, bcl‐2, and the proapoptosis protein, bax. The change in the expression ratio of bax and bcl‐2 determines whether apoptosis occurs (Wu et al., 2015). Caspases play essential roles in regulating the apoptosis induced by oxidative stress. Caspase‐3 is an important effector in the apoptotic process. Cleaved‐caspase‐3 is the least common of the apoptotic cells. During the process of induced apoptosis, activation of the initiator caspase‐8 can transmit death signals through the direct activation of the effector caspase‐3, and caspase‐9 is an initiator of caspase‐3 in the mitochondria‐dependent pathway (Hwang et al., 2006; Li et al., 2015). Therefore, cleaved‐caspase‐3/‐8/‐9 can be used as a reliable indicator to determine the severity of apoptosis (Li, Li, et al., 2017; Li, Lin, et al., 2017). In the current study, compared with the blank group, ethanol increased the expression of bax and c‐caspase‐3/‐8/‐9 but reduced the expression of bcl‐2. In addition, SHP‐1 and SHP‐2 could markedly induce a decrease in bax and c‐caspase‐3/‐8/‐9 levels and an increase in bcl‐2 expression compared with the control group (Figure 3a,b). In short, SHP inhibits caspase‐dependent apoptosis.

The mitochondria are responsible for most ATP production in the cell (Loperena & Harrison, 2017). Excessive ROS may result in changes of MMP and cause apoptosis (Rodenak‐Kladniew et al., 2018). In this study, ethanol treatment for 24 hr reduced the MMP, whereas SHP‐1 and SHP‐2 prevented the loss of MMP (Figure 3c,d). These results reveal that a decrease in MMP is likely involved in ethanol‐induced apoptosis.

DNA fragmentation is a hallmark of apoptosis (Han et al., 2015). A single‐cell gel electrophoresis (SCGE) assay, also known as comet assay, has been widely used to detect DNA damage (Bai et al., 2015). A comet assay is convenient for quantitatively studying concentration‐response relationships in vitro (Zhang et al., 2011). Furthermore, the length of the comet tail represents the extent of DNA damage (Ghazi et al., 2017). As demonstrated in Figure 3e,f, a significant increase in the length of the comet tail was observed in the HepG2/CYP2E1 cells exposed to ethanol. The length of the comet tails decreased with increasing concentrations of SHP‐1 and SHP‐2 compared with the control group. The results indicate that SHP reduced DNA damage.

NF‐E2‐related factor 2 (Nrf2), a transcription factor, regulates important antioxidant proteins, including heme oxygenase‐1 (HO‐1; Roh et al., 2018). Nrf2 exists in the cytoplasm by combining with the kelch‐like ECH‐associated protein 1 (keap1) under normal circumstances (Kašuba et al., 2017). In an oxidative stress environment, keap1’s active site cysteine residues are oxidized, preventing keap1 from interacting with Nrf2 (Li et al., 2018). Subsequently, Nrf2 binds to the antioxidant response element (ARE) to suppress oxidative stress (Zhang et al., 2017). Furthermore, HO‐1 may confer cytoprotection and preserve anti‐inflammatory, antiproliferative, and antiapoptotic functions by regulating oxidative stress (Schipper et al., 2019). In this study, the expressions of Nrf2 and HO‐1 were up‐regulated after pretreatment with SHP‐1 and SHP‐2 compared with the control group (Figure 4a,b). The results suggested that SHP could protect HepG2 cells against ethanol‐induced oxidative damage via the activation of Nrf2/HO‐1, which is consistent with the previous study (Li, Li, et al., 2017; Li, Lin, et al., 2017).

A series of studies have demonstrated that the Akt signaling pathway is associated with apoptosis in various cell types (Wang et al., 2014). Akt can also accelerate the activation of downstream nuclear factor‐κB (NF‐κB) and then regulate the apoptosis‐related kinase, bax/bcl‐2 (Qi et al., 2017). NF‐κB is involved in proliferation, differentiation, and apoptosis. In most cell types, NF‐κB is in the cytoplasm bound to IκB‐α, which is its inhibitory protein. IκB‐α may induce phosphorylation and degradation via the trimeric IκB kinase (IKK). The degradation of IκB‐α can free NF‐κB to enter the cell's nucleus and activate transcription of the target genes (Wang et al., 2013). In this study, Figure 4c–f shows that SHP had an antiapoptosis effect through inhibition of the Akt/NF‐κB pathways and the nuclear translocation of p65.

Apoptosis can be activated by signaling of mitogen‐activated protein kinases (MAPK; Wang et al., 2017). MAPKs contain extracellular signal‐related kinases (ERKs), stress‐activated p38 kinases, and c‐jun NH2‐terminal kinases (JNKs; Li, Li, et al., 2017; Li, Lin, et al., 2017). Generally, ERK1/2 is closely associated with cell growth and survival, whereas JNK and p38 mainly regulate oxidative stress and cell apoptosis. It has been demonstrated that ROS can activate the MAPK pathway and that attenuation of ROS by ROS scavengers can deactivate the MAPK signal transduction pathway (Zhang et al., 2018). In this study, Figure 4g,h indicates that ethanol activated the MAPK pathway, which led to HepG2 cell apoptosis. SHP‐1 decreased the expressions of p‐ERK, and p38 and SHP‐2 reduced the expressions of p‐JNK, p‐ERK, and p‐38. These results demonstrate the beneficial effects of SHP in HepG2 cells by regulating the MAPK signaling pathway to improve the antiapoptotic response.

Antioxidant capacity is related to the molecular size of the peptides, their amino acid composition, sequence, structure, and hydrophobic character. Hydrophobic amino acid residues (Ala, Val, Leu, Ile, Pro, Phe, Trp, Met, and Gly) have an antioxidant capacity because they can quench free radicals due to the abundance of their electrons (Coelho et al., 2019). Moreover, Gly residue can make the peptide skeleton more flexible, and its single hydrogen atom can serve as a proton‐donating to neutralize free radicals (Yang et al., 2019). In this study, SHP‐1 contained hydrophobic amino acids residues (Ala, Val, and Ile), and SHP‐2 contained hydrophobic amino acids residues (Ala, Gly, and Pro), which contributed to their antioxidant capacity. Aromatic amino acid residues (Phe and Tyr) can donate protons to electron‐deficient radicals to keep their ROS stable and enhance the potency of their radical scavenging capacity (Yang et al., 2019). Peptides containing amino acids with a phenolic hydroxyl group are efficient radical scavengers (Coelho et al., 2019). Furthermore, polar amino acid residues (Glu, Asp, and Lys) played a critical role in antioxidant activity, including metal ion chelating and hydroxyl radical scavenging activities. Therefore, polar amino acid residues, including Asp and Lys in SHP‐1 and Glu in SHP‐2 could play a critical role in antioxidant capacity. According to the above analysis and the results obtained from this study, the protective effects of SHP‐1 on ethanol‐induced oxidative stress in HepG2/CYP2E1 cells may be better than those of SHP‐2 from the aspect of an antioxidant. Based on the Western blot results and the molecular docking study, compared with SHP‐1, SHP‐2 may have a more obvious antiapoptosis effect.

## 5 CONCLUSIONS

In conclusion, this study demonstrated that ethanol can cause oxidative damage and apoptosis to HepG2/CYP2E1 cells. SHP‐1 and SHP‐2 decreased the production of ROS, the activity of GGT, and TNF‐α level. They also enhanced the activities of SOD and GSH and the MMP and reduced DNA damage. The protective effects of two SHPs against ethanol‐induced oxidative stress in HepG2 cells may be associated with the Nrf2/HO‐1, Akt/NF‐κB, and MAPK pathways. The administration of SHP protected cells against oxidative damage through antioxidant and antiapoptosis in two ways. These novel and interesting findings suggest that SHP could be beneficial for the prevention and treatment of ALD.

## CONFLICT OF INTEREST

The authors declare no competing financial interest.

## Data Availability

Research data are not shared.
